# Exercise training improves circulatory dynamics in adolescents with postural orthostatic tachycardia syndrome

**DOI:** 10.3389/fped.2025.1573842

**Published:** 2025-06-05

**Authors:** Yoshitoki Yanagimoto, Yuko Ishizaki, Toshiki Terashima, Ryuhei Yoshida, Kento Ishitani, Kohei Haraguchi, Mana Yamamoto, Mayumi Kubota, Yuto Adomi, Shinobu Yamasaki, Toshimitsu Suga, Kazunari Kaneko

**Affiliations:** ^1^ Department of Pediatrics, Kansai Medical University, Osaka, Japan; ^2^ Department of Health Science Center, Kansai Medical University, Osaka, Japan; ^3^ Department of Rehabilitation Medicine, Kansai Medical University, Osaka, Japan

**Keywords:** adolescents, circulatory dynamics, endurance exercise, exercise training, postural orthostatic tachycardia syndrome

## Abstract

**Introduction:**

Exercise training is recommended for PoTS; however, very few studies have examined the effectiveness of exercise training in young adolescents with PoTS. We evaluated the effects of ergometer endurance exercise on the circulatory dynamics of children with PoTS using cardiopulmonary exercise (CPX) testing, standing tests, and cardiac output monitoring.

**Methods:**

Overall, 28 participants with PoTS (19 males) aged 12–15 years were admitted to the Department of Pediatrics, Kansai Medical University General Medical Center, for 1 month between August 2020 and November 2023. Of the participants, 17 were assigned to the exercise group (13 boys) and 11 were assigned to the control group (6 boys). All participants underwent the standing test and CPX testing upon admission. The exercise group performed ergometer exercise for 30 min once per day, five times per week for 4 weeks. After 4 weeks, both groups completed the standing and CPX tests again. During the standing test, the patients underwent non-invasive hemodynamic monitoring using the AESCULON Mini®.

**Results:**

There were no significant differences between the two groups in demographic characteristics at admission (before the start of exercise training). Stroke volume, cardiac output, cardiac index, and thoracic fluid content increased after exercise training in the exercise group [pre- vs. post-exercise: cardiac output (ml) 61.7 vs. 73.1 (*P* = 0.009); cardiac output (L/min): 6.6 vs. 7.7 (*P* = 0.001); cardiac index (L/min/m^2^): 4.3 vs. 5.0 (*P* = 0.029); thoracic fluid content: 28.7 vs. 33.8 (*P* = 0.001)]. Exercise duration and maximal oxygen uptake (VO_2_) increased after exercise training in the exercise group on CPX testing [pre- vs. post-exercise: load time (min): 1.8 vs. 9.6 (*P* = 0.002), peak VO_2_ (ml/min/kg): 30.3 vs. 33.2 (*P* = 0.005)]. The hemodynamic and CPX test results were unchanged in the control group. No significant changes were observed in orthostatic test results in either group.

**Discussion:**

Endurance exercise training for 4 weeks increased cardiac output during orthostasis in children with PoTS and inhibited the downward migration of blood. We conclude that ergometer exercise training for 4 weeks in young adolescents with PoTS may improve circulatory dynamics during orthostasis.

## 1 Introduction

Postural orthostatic tachycardia syndrome (PoTS) is a complex, multi-system, chronic disorder of the autonomic nervous system that is characterized by orthostatic intolerance with an excessive heart rate (HR) increase and symptoms on standing, while blood pressure is maintained (1). PoTS is common in adolescents and young adults (1, 2), most commonly occurring at around 14 years of age (2), but the cause of the syndrome is not entirely clear (1). The main symptoms of PoTS are general fatigue, low energy, headache, cognitive impairment, muscle fatigue, chest pain, non-specific generalized weakness, dizziness, palpitations, presyncope, difficulty concentrating, and gastrointestinal symptoms (2–4). PoTS interferes with the daily lives of adolescents, influencing their ability to attend school due to its symptoms, and reduces quality of life (2, 5, 6). In addition, childhood PoTS increases the onset of depression and anxiety, both in the individual and among their family members, when physical symptoms persist (7). Moreover, economic damage occurs when the onset of PoTS coincides with the beginning of the individual's education or early career (8).

Three subtypes of PoTS, including hyperadrenergic, neuropathic, and hypovolemic PoTS, have been described. Although the pathogenesis of PoTS is heterogeneous and largely remains unclear (1, 5, 9, 10), these subtypes have some overlapping characteristics (1, 5, 11). Garland et al. (12) proposed that PoTS may simply be a final common pathway for several interrelated pathophysiologic mechanisms.

Deconditioning is considered to be closely related to PoTS (13, 14). Deconditioning is defined as reversible changes or loss of function in body systems owing to physical inactivity (15). This may cause myocardial atrophy, decreased cardiac output, decreased circulating plasma volume, and lower-extremity muscle atrophy, exacerbating circulatory ataxia during orthostasis (11). Deconditioning is also considered to be the final pathway in the pathogenesis of PoTS, as well as a factor involved in its pathogenesis (12–14, 16–19). Many teenage patients with PoTS experienced deconditioning; however, deconditioning is not the sole cause of tachycardia in PoTS (20, 21), and prolonged bed rest can exacerbate orthostatic intolerance, including PoTS (22).

A vicious cycle of reduced activity due to symptoms of PoTS and deconditioning occurs, which further exacerbates PoTS (10, 23).

Exercise training is already recommended as a treatment for PoTS, especially for its prevention and for the improvement of deconditioning (1, 4). According to its pathophysiology, exercise training is particularly effective in patients with hypovolemic PoTS (10). Several studies have shown that a progressive exercise program for patients with PoTS improves quality of life, increases stroke volume, and reduces orthostatic HR (13, 18, 24–27). However, few studies have evaluated the effectiveness of exercise training in adolescents with PoTS; most of the previous studies involved individuals aged ≥18 years, including adults aged >20 (17, 18, 24, 27, 28), only one study included individuals aged 15–18 (25), and no studies have targeted those aged <15 year (early teens). Furthermore, some patients have difficulty implementing exercise in their daily lives due to reduced orthostatic tolerance, so it is useful to evaluate the effectiveness of exercise training in adolescents with PoTS and to develop and disseminate a workable exercise program.

In the present study, we developed and implemented an exercise training program for young adolescents with PoTS that can be performed in either the supine or semi-supine position. We non-invasively evaluated the effects of this exercise program on circulatory dynamics to clarify the effectiveness of exercise training for PoTS in young adolescents.

## 2 Materials and methods

### 2.1 Participants

Twenty-eight junior high school students (19 boys and 9 girls) who were diagnosed with PoTS and who were hospitalized at the Department of Pediatrics, Kansai Medical University Medical Center, for 4 weeks between August 2020 and November 2023 were included. The diagnostic criteria for PoTS were an increment in HR of no less than 40 bpm within 10 min of active standing or head-up tilt and duration of symptoms was at least 3 months (1). Mean periods from onset was 14.6 months and those from diagnosis and start of treatment was 11.3. Out of the participants, 17 were diagnosed by the active standing test and another 11 by the head-up-tilt at the first diagnosis in our hospital. In addition, all participants were subjected to the active standing test on admission, and they met the diagnostic criteria for PoTS (*Δ*HR ≥40). The indications for admission to our hospital were “not attending school”, “lost the rhythm of life and unable to regain it at home”, and “rarely left the house”, which continued for over 1 month. All participants had no special medical history or comorbidities, such as hypermobile Ehlers-Danlos syndrome. The admissions in odd and even months were assigned to the exercise and control groups, respectively. Of the 28 participants, 17 (13 boys and 4 girls) completed the exercise training (exercise group), whereas 11 (6 boys and 5 girls) did not (control group).

### 2.2 Protocol

The participants underwent cardiopulmonary exercise (CPX) testing and the standing test upon admission. The load with a respiratory quotient of 1.15 in CPX testing was applied as reference, and the anaerobic threshold was measured (29). In the exercise group, the initial exercise intensity was set based on the load value 1 min before the appearance of the anaerobic threshold point as the exercise. The exercise group undertook recumbent ergometer exercise training in the supine position for 30 min once per day, five times per week, for a total of 4 weeks. Exercise training was performed in the semisupine position from week 3, and the intensity was increased by 10 W at the participant's request.

All participants received lifestyle guidance, including guidance to consume 2 L of water per day and to resume their regular lifestyle, in accordance with the orthostatic dysregulation diagnosis and treatment guidelines of the Japanese Society of Pediatric Psychosomatic Medicine (30). In addition, the participants attended an in-hospital classroom for long-term in-patient children five times per week on weekdays and lived a life similar to that of a normal school day. Both groups lived the same lifestyle except for the exercise training program.

### 2.3 Measurements

**Figure 1:**
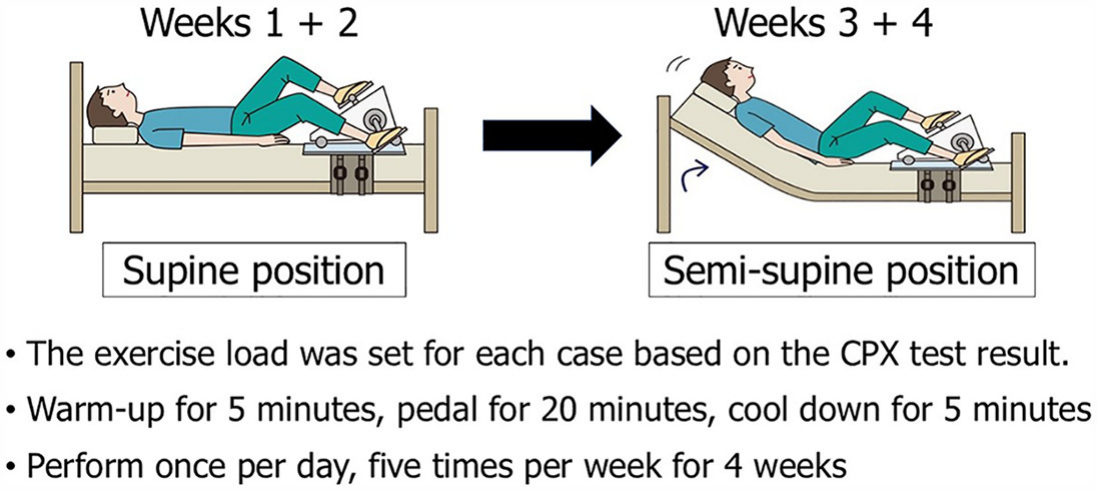
Protocol for exercise training. CPX, cardiopulmonary exercise.

The exercise training program involved 30 min of ergometer exercise training once per day, five times per week for 4 weeks. The initial exercise load was determined based on the CPX test results at admission (29). Recumbent ergometer training was started in the supine position while in bed, and it was performed in the semi-supine position from week 3, increasing in intensity as exercise tolerance increased (Figure 1). Four weeks later, both groups again performed the standing test and CPX testing. Circulatory dynamics were non-invasively monitored using the AESCULON Mini® during the standing test.

#### 2.3.1 CPX testing

All patients performed CPX testing using a cycle ergometer. After 5 min of rest on the ergometer (75XL II ME; Combi Co., Ltd., Tokyo, Japan), the exercise began with a warm-up at 20 W for 4 min. Following the warm-up, the load was gradually increased by 10 W/minute for patients of standard fitness, and by 5–10 W/minute for patients of low fitness. A cool-down exercise for a minimum of 5 min until subjective symptoms and vital signs stabilized. Twelve-lead electrocardiogram was used for monitoring during CPX testing to continuously monitor the HR and the occurrence of arrhythmia. Blood pressure was measured every minute during exercise. Breath gas analysis was performed using a breath-by-breath method with an expired gas analyzer (AE-310S; Minato Medical Science Co., Ltd., Osaka, Japan). The anaerobic threshold was determined using the V-slope method. Peak oxygen uptake (VO_2_) and HR were defined as the peak values achieved during incremental exercise. The termination criterion was achievement of the symptom limit or fulfillment of the criteria for discontinuing the exercise test. The duration that each participant was able to continue exercise was recorded as the load time.

#### 2.3.2 Standing test

Standing test was performed in accordance with the orthostatic dysregulation diagnosis and treatment guidelines of the Japanese Society of Pediatric Psychosomatic Medicine (30). Before the standing test, the participants were required to rest for 10 min. Then, blood pressure and HR were measured three times every minute. Next, the participants stood up and remained standing for 10 min. Blood pressure and HR were measured every minute after standing using a mercury-free automatic blood pressure monitor (KM-385OD® KENZMEDICO Co., Ltd., Saitama, Japan).

#### 2.3.3 Circulatory dynamics

Hemodynamic evaluation during the standing test was performed using the AESCULON Mini ® (Osypka Medical GmbH, Stuttgart, Germany). The AESCULON Mini is a device that can quickly and non-invasively determine hemodynamic status using electrical cardiometry. Two sensors are attached to the left side of the neck and two to the left side of the chest. Current flows from the outer sensor and passes through the path of least resistance (aorta filled with blood). The inner sensor measures the change in current. Each heartbeat changes the volume and velocity of blood in the aorta and simultaneously changes the orientation of the red blood cells. By measuring the change in conductivity due to this change in red blood cell orientation, hemodynamic indices can be measured simply and non-invasively (31). The AESCULON Mini can be used from newborns to adults (31). In addition, few papers have reported experience with its use for hemodynamic evaluation in teenagers (32, 33).

#### 2.3.4 Exercise training

The exercise training program is illustrated in Figure 1. An ergometer was attached to the bed, and the participant underwent exercise training in the supine position for the first 2 weeks. A 5-min warm-up was performed, followed by 20 min of pedaling exercise at the set exercise load, followed by a 5-minute cool-down period. This was performed once per day for five days per week. During the latter two weeks (weeks 3 + 4), the bed was raised and the exercise was performed in the semi-supine position. If possible, the exercise load was increased.

### 2.4 Statistical analysis

Statistical analysis was performed using SPSS, version 29. The groups were compared using Fisher's exact test, the Kruskal–Wallis test, or the Mann–Whitney *U*-test, as appropriate, and within-group comparisons at the time of admission and 4 weeks later were performed using Wilcoxson's rank-sum test.

### 2.5 Ethics

The participants were informed of the purpose of the study, both orally and in writing, and written informed consent was obtained from all participants. The study was approved by the Ethics Review Committee of Kansai Medical University Medical Center (approval number: 2020036).

## 3 Results

### 3.1 Baseline demographic characteristics

All 28 participants completed the study. All 17 participants in the exercise group completed the 4-week exercise program. No significant differences were found in the baseline demographic characteristics, periods from disease onset, diagnosis, and start of treatment, and volume of oral water intake between the two groups (Table 1). On admission, 52.9% of the patients (*n* = 9) in the exercise group were taking alpha-stimulants, 23.6% (*n* = 4) were taking beta-blockers, and 5.9% (*n* = 1) were taking droxidopa. In the control group, 54.5% (*n* = 6) were taking alpha-stimulants. No significant difference in medication use was noted between the two groups (Table 1). None of the patients changed their medications during hospitalization.

**Table 1:** Participants' demographic characteristics.

Demographic characteristics	Participants			*P* value
	Exercise training (*N* = 17)	Control (*N* = 11)	Total (*N* = 28)	
Age, years (SD)	13 (0.8)	14 (0.6)	13.5 (0.7)	0.932^a^
Range	12–15	13–15	12–15	
Sex, *n* (%)				0.489^b^
Female	4 (23.6)	5 (45.5)	9 (32.1)	
Male	13 (76.4)	6 (54.5)	19 (67.9)	
Height, cm (SD)	161.9 (7.5)	157.3 (6.2)	160.2 (7.4)	0.508^a^
Weight, kg (SD)	45.2 (2.3)	43.2 (5.3)	44.4 (3.8)	0.861^a^
BMI (SD)	19.2 (3.2)	18.7 (2.7)	19.0 (3.0)	1^a^
Amount of oral intake water, ml/day (SD)				
Admission	1,396 (836)	1,209 (483)	1,323 (724)	0.99^a^
4 weeks later	1,534 (944)	1,309 (755)	1,446 (882)	0.733^a^
Medications *n* (%)				
Beta blocker	4 (23.6)	0 (0)	4 (14.3)	0.216^a^
Alpha agonist	9 (52.9)	6 (54.5)	15 (53.6)	1^a^
Doloxidopa	1 (5.9)	0 (0)	1 (3.6)	1^a^
None	7 (41.2)	5 (45.5)	12 (42.9)	1^a^
Periods, months (SD)				
From onset of disease	13.9 (7.7)	14.6 (6.0)	14.6 (7.0)	0.937^a^
From diagnosis and start of treatment	10.6 (6.2)	12.5 (6.6)	11.3 (6.3)	0.797^a^

BMI, body mass index; SD, standard deviation. Data are expressed as the mean.

^a^ Kruskal-Wallis *p* value.

^b^ Fisher's exact *p* value.

### 3.2 Circulatory dynamics

There were no significant differences in the results of all parameters at the time of admission (before the start of exercise training) between the two groups (Table 2). In the pre- and post-exercise training comparisons, stroke volume, cardiac output, cardiac index, thoracic fluid content, and contractility index in the upright position in the exercise group increased after exercise training [pre- vs. post-exercise: stroke volume (ml): 61.7 vs. 73.1 (*P* = 0.009); cardiac output (L/min): 6.6 vs. 7.7 (*P* = 0.001); cardiac index (L/min/m^2^): 4.3 vs. 5.0 (*P* = 0.029); thoracic fluid volume: 28.7 vs. 33.8 (*P* = 0.001); contractility index: 70.0 vs. 81.2 (*P* = 0.004)]. In the control group, intravascular corrected flow time (ms), which indicates intravascular blood flow, was lower after 4 weeks than at admission (297.8 vs. 305.5, *P* = 0.026). There were no significant changes in other parameters in either the supine or upright positions (Table 3).

**Table 2:** Results of circulatory dynamics, CPX testing, and the standing test at admission.

Inspection items	Participants(*N* = 28)		*P* value^a^
	Exercise training (*N* = 17)	Control (*N* = 11)	
Hemodynamic index			
Supine position			
Heart rate, bpm	70.7	66.9	0.404
Stroke volume, ml	70.2	68.6	0.329
Cardiac output, L/min	4.9	4.5	0.771
Cardiac index, L/min/m^2^	3.3	3.1	0.677
Thoracic fluid content	31	31.8	1
Flow time corrected, ms	317.9	320.7	0.378
Stroke volume variation, %	9.6	9.1	0.89
Index of contractility	73.9	74	1
Systolic time ratio	0.37	0.35	0.244
Uplight position			
Heart rate, bpm	107.8	102.6	0.578
Stroke volume, mL	61.7	61	0.853
Cardiac output, L/min	6.6	6.2	0.458
Cardiac index, L/min/m^2^	4.3	4.2	0.926
Thoracic fluid content	28.7	29.2	0.963
Flow time corrected, ms	291.9	305.5	0.191
Stroke volume variation, %	15.4	13.2	0.147
Index of contractility	70	68.49181818	0.711
Systolic time ratio	0.57	0.55	0.711
CPX testing			
First HR, bpm	85.1	90.1	0.264
First SBP, mmHg	115.6	112.4	0.547
First DBP, mmHg	67.5	67.6	0.926
LoadTime, min	9.6	9.1	0.746
Final HR, bpm	176.3	179.9	0.963
Final SBP, mmHg	154.6	154.1	0.853
Final DBP, mmHg	66.3	72.6	0.329
Peak VO_2_, mL/min/kg	30.3	29.8	0.781
HRR, sec	29.6	27	0.378
Standing test			
Max heart rate, bpm	114.3	110.6	0.853
Change of heart rate, bpm	49.5	48	0.89
Change of systolic blood pressure, mmHg	−2	−1.6	1

CPX, cardiopulmonary exercise; DBP, diastolic blood pressure; HR, heart rate; SBP, systolic blood pressure; peak VO_2_, peak pxygen uptake; HRR, heart rate recovery time.

^a^ Mann-Whitney U test *p* value.

**Table 3:** Comparison between the results at the time of admission and 4 weeks later.

Inspection items	Participants (*N* = 28)					
	Exercise training (*N* = 17)			Control (*N* = 11)		
	Admission	4 weeks later	*P* value^a^	Admission	4 weeks later	*P* value^a^
Hemodynamic index						
Supine position						
Heart rate, bpm	70.1	69.5	0.831	66.8	70.1	0.131
Stroke volume, ml	70.2	79.5	0.21	68.6	72.6	0.534
Cardiac output, L/min	4.9	5.5	0.193	4.5	5	0.286
Cardiac index, L/min/m^2^	3.3	3.6	0.17	3.1	3.4	0.248
Thoracic fluid content	31	35.7	0.058	31.8	33	0.722
Flow time corrected, ms	317.9	322.5	0.193	320.7	321.3	0.534
Stroke volume variation, %	9.6	10.1	0.636	9.1	9.4	0.45
Index of contractility	73.9	77.8	0.586	73.981818	73.21363636	0.929
Systemic vascular resistance, dyns/cm^5^	1321.2	1275.6	0.619	1520.1	1272.3	0.091
SVR index	2003.6	1922.5	0.523	2172.7	1865	0.091
Uplight position						
Heart rate, bpm	107.8	108.9	0.554	102.6	107.4	0.075
Stroke volume, ml	61.7	73.1	0.009	61	60.6	0.248
Cardiac output, L/min	6.6	7.7	0.001	6.2	6.4	0.79
Cardiac index, L/min/m^2^	4.3	5	0.001	4.2	4.3	0.79
Thoracic fluid content	28.7	33.8	0.029	29.2	30.1	0.79
Flow time corrected, ms	291.9	294.2	0.653	305.5	297.8	0.026
Stroke volume variation, %	15.4	14.8	0.795	13.2	14.9	0.374
Systemic vascular resistance, dyns/cm^5^	70	81.2	0.004	68.491818	65.90916667	0.534
SVR index	0.57	0.57	0.943	0.53	0.55	0.859
CPX testing						
First time: admission						
Pre HR, bpm	85.1	84.9	0.776	90.4	88.5	0.562
Pre SBP, mmHg	115.6	112.2	0.205	112.4	108.2	0.398
Pre DBP, mmHg	67.5	60.6	0.061	67.6	63.7	0.325
LoadTime, min	9.6	10.8	0.002	9.1	9.5	0.157
Last HR, bpm	176.3	179.8	0.265	179.9	183.4	0.239
Last SBP, mmHg	154.6	148.9	0.061	154.1	157.8	0.374
Last DBP, mmHg	66.3	63.8	0.887	72.6	72.5	0.76
Peak VO_2_, mL/min/kg	30.3	33.2	0.005	29.8	30.5	0.645
HRR, sec	29.6	31.5	0.442	27	33.7	0.83
Standing test						
Max HR, bpm	114.3	118.3	0.345	110.6	113.2	0.102
*Δ* HR, bpm	49.5	53.6	0.492	48	45.5	0.61
*Δ* SBP, mmHg	−2	−2.2	0.534	−1.6	−0.7	0.331
Amount of oral intake water, mL/day	1396.5	1534.1	0.283	1209.1	1309.1	0.82

CPX, cardiopulmonary exercise; DBP, diastolic blood pressure; HR, heart rate; peak VO_2_, peak oxygen uptake; SBP, systolic blood pressure; SVR, systemic vascular resistance.

^a^ Wilcoxon's rank sum test.

### 3.3 CPX testing

There were no significant differences in the CPX test results at admission (before the start of exercise training) between the two groups (Table 2). Load time, which is the exercise duration, and peak VO_2_ increased in the exercise group after 4 weeks [pre- vs. post-exercise: load time (min): 9.6 vs. 10.8 (*P* = 0.002), peak VO_2_ (ml/min/kg): 30.3 vs. 33.2 (*P* = 0.005)] (Table 3). The control group demonstrated no significant change in these two test results (Table 3).

### 3.4 Standing test

There were no significant differences in the standing test results at admission (before the start of exercise training) between the two groups (Table 2). Comparison of the results between the two groups at admission and 4 weeks later showed no significant differences in blood pressure reduction, HR elevation, or maximum HR (Table 3).

## 4 Discussion

### 4.1 Efficacy of exercise training

This study revealed that ergometer exercise training in the supine position for 4 weeks improved the circulatory dynamics of young adolescents with PoTS. Several studies evaluating the effects of exercise training on PoTS (Table 4) have reported that exercise training improves orthostatic test results, including tachycardia during orthostasis (12, 13, 18, 25–28); circulatory dynamics, including increasing cardiac output and circulating plasma volume (13, 26); symptoms (24, 27, 28); and quality of life (13). However, all of these reports were conducted in young adults or older adolescents, making our study the first to evaluate the effects of endurance exercise training in young adolescents with PoTS.

**Table 4:** Details of the previous studies’ design, participant characteristics, designation to exercise training, and result.

Reference	Design	Study group		Training		Results
		Participant	Age, years	Duration, months	Kind of training	
Winker, et al. 2005	RCT	PoTS *n* = 31 (exerxixe training *n* = 16, control *n* = 15 )	18–23	3	Endurance	Symptom improvement, increase in HR on standing test
Fu, et al. 2010	Prospective cohort study	PoTS *n* = 27 (exercise training *n* = 27)	21–33	3	Endurance and resistance	Increase in blood volume and left ventricular mass, increase in HR on standing test, improvement in QoL
		Healthy control *n* = 16 (No exercise training *n* = 16)	23–35			
Fu, et al. 2011	RCT	PoTS *n* = 19 (exercise training+propranorol *n* = 9, exercise training+placebo *n* = 10)	18–36	3	Endurance and resistance	Increase in stroke volume and cardiac output, improvement in reaction to RAA, improvement in QoL
		Healthy control *n* = 15 (No exercise training *n* = 15)	21–41			
Galbreath, et al. 2011	Prospective cohort study	PoTS *n* = 17 (exercise training *n* = 17)	18–36	3	Endurance and resistance	Improvement in arterial-cardiac baroreceptor reflex sensitivity, improvement in tachycardia upon standing
		Healthy control *n* = 17 (No exercise training *n* = 17)	21–41			
Shibata, et al. 2012	Prospective cohort study	PoTS *n* = 19 (exercise training *n* = 19 )	25–29	3	Endurance and resistance	Decrease in HR during exercise training, increase in stroke volume and blood volume
		Healthy control (*n* = 10); no exercise training (*n* = 10)	28–32			
George, et al. 2016	Prospective cohort study	PoTS *n* = 251 (exercise training *n* = 251)	15–37	3	Endurance and resistance	Improvement in tachycardia on standing test
Gibbons, et al. 2021	Prospective cohort study	PoTS *n* = 77 (exerxixe training *n* = 48, control *n* = 29)	19–32	6	Endurance	Improvement in tachycardia on standing test, reduced syncope
Weatley-Guy, et al., 2023	Prospective cohort study	PoTS *n* = 49 (exerxixe training *n* = 26, control *n* = 23)	18–66	3	Endurance	Increase in peak VO_2_, increased orthostatic tolerance
Present study	Prospective cohort study	PoTS *n* = 28 (exerxixe training *n* = 17, control *n* = 11)	12–15	1	Endurance	Increase in stroke volume and cardiac output upon standing, increase in TFC

HR, heart rate; PoTS, postural tachycardia syndrome; RCT, randomized controlled trial; QoL, quality of life, RAA, renin angiotensin aldosterone system; TFC, thoracic fluid content.

Although the 2015 consensus recommends exercise training as a treatment for PoTS with the highest recommendation (Class IIa), in clinical practice, some patients have difficulty performing exercise in the standing position. This is because of the difficulty they experience with standing. Fu et al. and Stewart et al. suggested exercises that can be performed in the supine position (11, 16). Specifically, Fu et al. recommended recumbent biking, swimming, and rowing as specific exercises for PoTS (16). Of these, we considered recumbent (supine) ergometer training to be the easiest to implement and sustain in daily life, making it a worthwhile exercise to evaluate in this study.

Based on the suggestions of other scholars (11, 16), this study created and implemented an exercise regimen in young adolescents that incorporated ergometer exercise training in the supine position, which resembles exercise on a recumbent bike. Adolescents with PoTS received 4 weeks of in-patient care for the purpose of daily living rehabilitation at our hospital. We were able to compare the effects of exercise training by obtaining data for both groups, and the participants in each group had almost the same living environment. The only difference was that exercise training was incorporated into one group but not the other, which is a unique aspect of this study.

Based on the results of this study, exercise training using an ergometer in the supine position is effective and should be recommended to young adolescents with PoTS (aged 12–15 years).

Although patients can perform exercise under the supervision of medical staff in the hospital setting, it is difficult to confirm whether they are still exercising at the same pace after hospital discharge. We proposed a viable exercise program for young patients with PoTS; however, the sustainability of this exercise program over a longer duration 3 months has been proposed in previous studies (12, 13, 18, 21, 22, 26, 28), should be evaluated in the future.

### 4.2 Effect of exercise training on the pathophysiology of PoTS

Exercise training is said to be effective for hypovolemic PoTS (10), one of the three main subtypes of PoTS, based on reports of improved circulating plasma volume with exercise training (26). In addition, deconditioning is closely related to hypovolemic PoTS. Deconditioning causes a decrease in circulating plasma volume and orthostatic tolerance, which are thought to underpin PoTS onset and exacerbation (19, 34). Promisingly, exercise training has been reported to improve deconditioning and PoTS (13, 35).

The results of this study suggest that exercise training improves myocardial function and circulating plasma volume by increasing cardiac output and cardiac index, improving the CPX test results. The increase in thoracic fluid volume during standing indicated an increase in venous pooling and a reduction in downward blood shift during standing, perhaps resulting from increased lower-extremity muscle strength with exercise training. The increase in both stroke volume and cardiac output in the assessment of cardiac performance, the increase in cardiac contractility index, and the increase in exercise tolerance according to the CPX test results suggest that exercise training improved cardiac function in this population.

In a previous study evaluating the effects of exercise training on cardiac performance, Fu et al. (13) and Shibata et al. (26) suggested that patients with PoTS had decreased cardiac output, which was improved by exercise, and this was attributed to improved circulating plasma volume (an increase of approximately 6%) and increased cardiac contractility. In addition, Fu et al. (13) reported that exercise training itself increased circulating plasma volume.

Similar to previous studies, stroke volume and cardiac output were increased by exercise training in the present study, suggesting that 1 month of exercise training in young adolescents with PoTS may increase circulating plasma volume and cardiac contractility. The increase in contractility and exercise capacity, exercise duration, and peak VO_2_ on CPX testing (i.e., the improvement in exercise tolerance) may have supported the improvement in cardiac function. We suggest that the improvement in cardiac function along with the increase in circulating plasma volume improved cardiac output and improved hypovolemic PoTS.

No previous reports have investigated the relationship between the effects of exercise training and thoracic fluid content in patients with PoTS. In the present study, thoracic fluid content during standing increased after exercise training. This result may suggest that exercise training reduces the downward shift in blood volume that occurs during orthostasis, or that venous return is more rapid once the downward shift has occurred. We suggest that this is due to a reduction in hypovolemia, as well as an improvement in the pumping function of the lower-extremity muscles resulting from pedaling exercise on the ergometer. Stewart et al. (36) reported excessive blood pooling in the veins of the lower extremities in pediatric patients with PoTS. Moreover, Jacob et al. attributed venous pooling to a disturbance in autonomic innervation in the peripheral vessels (37). This autonomic neuropathy in the peripheral blood vessels, which causes venous pooling and decreased cerebral blood flow and thoracic blood volume, is defined as neuropathic PoTS, one of the three main subtypes of PoTS (1, 4, 10). Resistance exercise training to improve venous pooling by improving lower-extremity muscle pump function has been proposed as a treatment for neuropathic PoTS (16). However, the results of the present study suggest that endurance exercise training may also improve the pump function of the lower-extremity muscles. We speculate that the improved pump function of the lower-extremity muscles is a result of increased lower-extremity muscle strength, even with endurance exercise training. This suggests that exercise training may be effective for improving not only hypovolemic PoTS, but also neuropathic PoTS, even if endurance exercise training is used alone. In addition, it is said that muscle weakness in the lower extremities due to deconditioning causes venous pooling (11), so exercise training may be considered to improve venous pooling by improving deconditioning.

No significant differences were noted in the results of the control group between admission and 4 weeks later. The indications for admission to our hospital included “not attending school”, “lost the rhythm of life and unable to regain it at home”, and “rarely left the house” for over 1 month. We suspect that the participants who met these criteria were already inactive prior to their admission. We believe that deconditioning due to reduced physical activity experienced by participants prior to their admission, due to PoTS symptoms, as well as PoTS itself, resulted in diminished circulatory dynamics in both groups prior to their admission. Although not accurately assessed, peak VO_2_ on admission was lower than expected for their age in both groups (38), suggesting that deconditioning had occurred (14, 21). As described in the Methods, the exercise and control groups lived the same lives during their hospitalization, except for 30 min of exercise for the exercise group. All participants were not confined to their beds throughout the day because they attended an in-hospital class within the ward during the day and participated in various activities. Thus, we believe that the inpatient life of the control group was not more restrictive than their usual home life; however, they unlikely engaged in sufficient intensity of activity to improve PoTS and deconditioning.

There were no significant differences in the results of the standing test between before and after exercise training in the present study. Several previous studies have reported that 3 months of exercise training improved the results of orthostatic tests, mainly demonstrated as an improvement in tachycardia during standing (13, 17, 28). The difference between our study and previous studies may be explained by the different durations of exercise training. Our study showed that the circulatory dynamics of PoTS improved after 4 weeks of exercise training, but the results of the standing test were unchanged. This result suggests that 4 weeks is not enough time to improve the results of the standing test, even though an improvement in circulatory dynamics was observed. Therefore, at least 3 months of continued exercise training may be needed to identify any improvement in the results of the standing test. Future studies are needed to evaluate the effects of exercise training over a longer period in this population. A recent study revealed significant improvement in both the maximum heart rate and heart rate increase during the active standing test 3 weeks after multifaceted treatment, including exercise training, in PoTS (39). This exercise training includes not only endurance exercise but also resistance exercise, muscle stretching, occupational therapy, and cognitive behavioral therapy. Although 1 month is not sufficient to improve tachycardia in PoTS by endurance exercise alone, as shown in the present results, and a minimum of 3 months is necessary, as previously reported. Moreover, endurance exercise combined with other exercises and therapies may improve tachycardia in PoTS within 1 month. Future studies should focus on comparing the endurance exercise group with a multiple exercise group which engages in endurance + resistance + stretching training.

### 4.3 Limitations

This study had some limitations that should be considered. First, although there was no significant difference between the two groups in the use of medication, we were unable to completely rule out the influence of medication in this study. However, none of the patients adjusted their medications during hospitalization, so we do not believe that medication would have significantly impacted the results. Second, a previous study showed that the symptoms of PoTS improved after 3 months of exercise training (27, 28), but we were unable to assess subjective symptoms in this study because the symptoms of PoTS are diverse and vary among patients. We believe that the establishment of a comprehensive evaluation method for the subjective symptoms of PoTS is an issue to be addressed in the future. Third, circulatory dynamics may have improved with other treatments (e.g., rehabilitation) during hospitalization. When this point is compared between the exercise group and the control group, there was no change in the control group, even within the county, so it can be assumed that the main improvement in circulatory dynamics was due to exercise training. Fourth, we were unable to consider the effect of the amount of water consumed and its changes over the course of the 4-week period, although there was no significant difference in the amount of water consumed between the two groups at the time of the examinations. The possibility remains that long-term consumption of water may have affected the circulating plasma volume and circulatory dynamics. Therefore, it cannot be ruled out that this may have influenced the results. During hospitalization, 2 L of fluid intake per day was recommended, but the daily fluid intake, including fluid in the diet, was not strictly measured. Closer monitoring of water consumption may be a useful consideration for future studies.

In addition, this study has several limitations regarding generalizability based on the participants.

First, the study participants were primarily composed of boys; however, approximately 80% of adolescents diagnosed with PoTS were girls (11). Our hospital specializes in the inpatient treatment of PoTS and eating disorders. Because eating disorders are more prevalent in girls, fewer beds are available for girls with PoTS than for their male counterparts. This likely led to a higher hospitalization rate for boys with PoTS, resulting in the high number of male participants in the study. We recognize the need to increase the number of female participants and reexamine the present results in future studies. Second, there may be a bias regarding the severity of participants’ condition because our hospital specializes in the inpatient treatment of PoTS, and many patients admitted exhibit severe PoTS. Third, given that most participants were from urban areas, the results may not apply to patients from nonurban areas where lifestyles vary. Furthermore, given the small sample size, future studies will need to increase the number of cases and revalidate the present results.

## 5 Conclusion

Four weeks of ergometer exercise training in the supine position improved the circulatory dynamics of young adolescents with PoTS. Establishment and dissemination of appropriate exercise training regimens may help to improve the prognosis and quality of life of this population.

## Data Availability

The original contributions presented in the study are included in the article/Supplementary Material, further inquiries can be directed to the corresponding author.
